# Structure-function analysis and therapeutic efficacy of antibodies to fungal melanin for melanoma radioimmunotherapy

**DOI:** 10.1038/s41598-018-23889-z

**Published:** 2018-04-03

**Authors:** J. D. Nosanchuk, A. Jeyakumar, A. Ray, E. Revskaya, Z. Jiang, R. A. Bryan, K. J. H. Allen, R. Jiao, M. E. Malo, B. L. Gómez, A. Morgenstern, F. Bruchertseifer, D. Rickles, G. B. Thornton, A. Bowen, A. Casadevall, E. Dadachova

**Affiliations:** 1 Albert Einstein College of Medicine, Bronx, New York USA; 2 University of Saskatchewan, Saskatoon, SK Canada; 3 School of Medicine and Health Sciences, Universidad Rosario, Bogota, Colombia; 4 European Commission, Joint Research Centre, Directorate for Nuclear Safety and Security, Karlsruhe, Germany; 5 RadImmune Therapeutics, Tarrytown, NY USA; 6 Pain Therapeutics, Inc, Austin, TX USA; 7 Johns Hopkins Bloomberg School of Public Health, Baltimore, MD USA

## Abstract

Metastatic melanoma remains difficult to treat despite recent approvals of several new drugs. Recently we reported encouraging results of Phase I clinical trial of radiolabeled with ^188^Re murine monoclonal IgM 6D2 to melanin in patients with Stage III/IV melanoma. Subsequently we generated a novel murine IgG 8C3 to melanin. IgGs are more amenable to humanization and cGMP (current Good Manufacturing Practice) manufacturing than IgMs. We performed comparative structural analysis of melanin-binding IgM 6D2 and IgG 8C3. The therapeutic efficacy of ^213^Bi- and ^188^Re-labeled 8C3 and its comparison with anti-CTLA4 immunotherapy was performed in B16-F10 murine melanoma model. The primary structures of these antibodies revealed significant homology, with the CDRs containing a high percentage of positively charged amino acids. The 8C3 model has a negatively charged binding surface and significant number of aromatic residues in its H3 domain, suggesting that hydrophobic interactions contribute to the antibody-melanin interaction. Radiolabeled IgG 8C3 showed significant therapeutic efficacy in murine melanoma, safety towards healthy melanin-containing tissues and favorable comparison with the anti-CTLA4 antibody. We have demonstrated that antibody binding to melanin relies on both charge and hydrophobic interactions while the *in vivo* data supports further development of 8C3 IgG as radioimmunotherapy reagent for metastatic melanoma.

## Introduction

Melanoma is a cancer with increasing incidence and is anticipated to affect roughly 73,380 new patients in the United States in 2016 and over 10,130 expected deaths this year^1^. While stage 1 and 2 melanoma can be surgically cured, the aggressive metastatic nature of this malignancy is responsible for a poor prognosis with an estimated 10-year survival of just 9% in patients with stage IV melanoma^2,3^. Until 2011, treatment options for patients with stage IV disease were limited and offered marginal improvement in overall survival. Approval by FDA of vemurafenib, which inhibits mutated B-RAF protein, offers hope for 40–60% melanoma patients carrying this mutation. Unfortunately, the spectacular initial responses of vemurafenib have been relatively short lived and are often followed by recurrences^4^. Efforts to restore latent anti-tumor immunity have focused on monoclonal antibody (mAb)-based interventions targeting CTL antigen 4 (CTLA-4)^5,6^ and programmed cell death protein 1 (PD-1) on T lymphocytes and its principal ligand (PD-L1) on tumor cells. Ipilimumab, an antibody targeting CTLA-4, appears to restore tumor immunity at the priming phase and prolongs overall survival in some patients, whereas anti-PD-1/PD-L1 antibodies restore immune function in the tumor microenvironment^7,8^. With only relatively few patients responding well to the currently available immune therapies along with the presence of very serious side effects and the high costs of immunotherapy^9,10^, there remains an urgent need for other approaches to combat metastatic melanoma which could work alone or in concert with immunotherapy.

In our search for new treatments for metastatic melanoma that would not rely on specific genotypes, biochemical pathways or the variability of an individual’s immune response, we turned to radioimmunotherapy (RIT) targeting melanin. RIT has emerged as a useful anti-tumor therapy via the targeting of radionuclides with antibodies against tumor-associated antigens^11^. RIT has historically proven successful in the treatment of primary, recurrent, and refractory non-Hodgkin’s lymphomas using anti-CD20 mAbs coupled to two β-emitters, Yttrium-90 and Iodine-131^12^. In the last decade the use of α-emitting radionuclides is gaining momentum both in clinical trials and in the preclinical studies which is driven by the advantages of α-emitters over β-emitters, including very specific targeting of the diseased cells due to the α-particles’ short 50–80 µm tissue range, and increased killing efficiency due to high linear energy transfer^13,14^.

Melanoma owes its name to the presence of the melanin pigment. Pigmentation is a very important factor in melanoma biology. The presence or absence of pigment affects melanoma patients survival, radiotherapy, chemotherapy and immune therapy, as was found in experimental models and in melanoma patient population^15–21^. Pigmentation is also related to hypoxic conditions and HIF-1α expression^22^, and plays a role in the regulation of physiological and pathological conditions^19^.

Historically, melanin was not considered a target for RIT because it is an intracellular pigment contained in organelles called melanosomes, and thus assumed not to be accessible to Ab. However, melanomas are rapidly growing tumors with high rates of cellular turnover and cell necrosis releases melanin into the extracellular space, where it is accessible to radiolabeled mAbs to melanin. Such antibodies deliver cytotoxic radiation to nearby malignant cells via the so called “cross-fire” effect. Furthermore, this strategy is attractive because melanin in normal tissues with very little cellular turnover is not accessible to mAbs by virtue of its “safe” intracellular location. Several IgM isotype mAbs to fungal melanin, such as 6D2 mAb, have been generated in our laboratories in the process of studying *Cryptococcus neoformans* melanogenesis *in vivo* and were shown to bind to melanins of different origins^23^. Experimental results have established the feasibility of targeting melanin released from dead melanoma cells in tumors with melanin-binding 6D2 mAb labeled with the beta-emitting radionuclide 188-Rhenium (^188^Re)^24,25^. Moreover, a phase Ia/Ib clinical trial of ^188^Re-6D2 mAb in patients with Stage III/IV melanoma demonstrated safety of RIT, and the trial data was indicative of its efficacy, as there was a prolongation in patients’ median survival^26^. However, the IgMs are difficult to produce under the cGMP (current Good Manufacturing Practice) conditions, to purify and to radiolabel. For this reason, the continuous development of RIT targeting melanin towards the clinical product calls for IgG isotype. IgGs are generally more amenable to pre-clinical development including chimerization or humanization and large scale manufacturing for clinical trials compared to mAbs with IgM isotype. More recently, our laboratories generated an IgG type mAb (8C3) to melanin from another pathogenic fungus, *Paracoccidioides brasiliensis*^27^. Here we report the results of the comparative structural investigation of the novel 8C3 IgG and the previous generation 6D2 IgM and 8C3 evaluation as a potential RIT agent in aggressive murine melanoma.

## Results

### IgM 6D2 and IgG 8C3 to melanin manifest similar V regions

**Figure 1:**
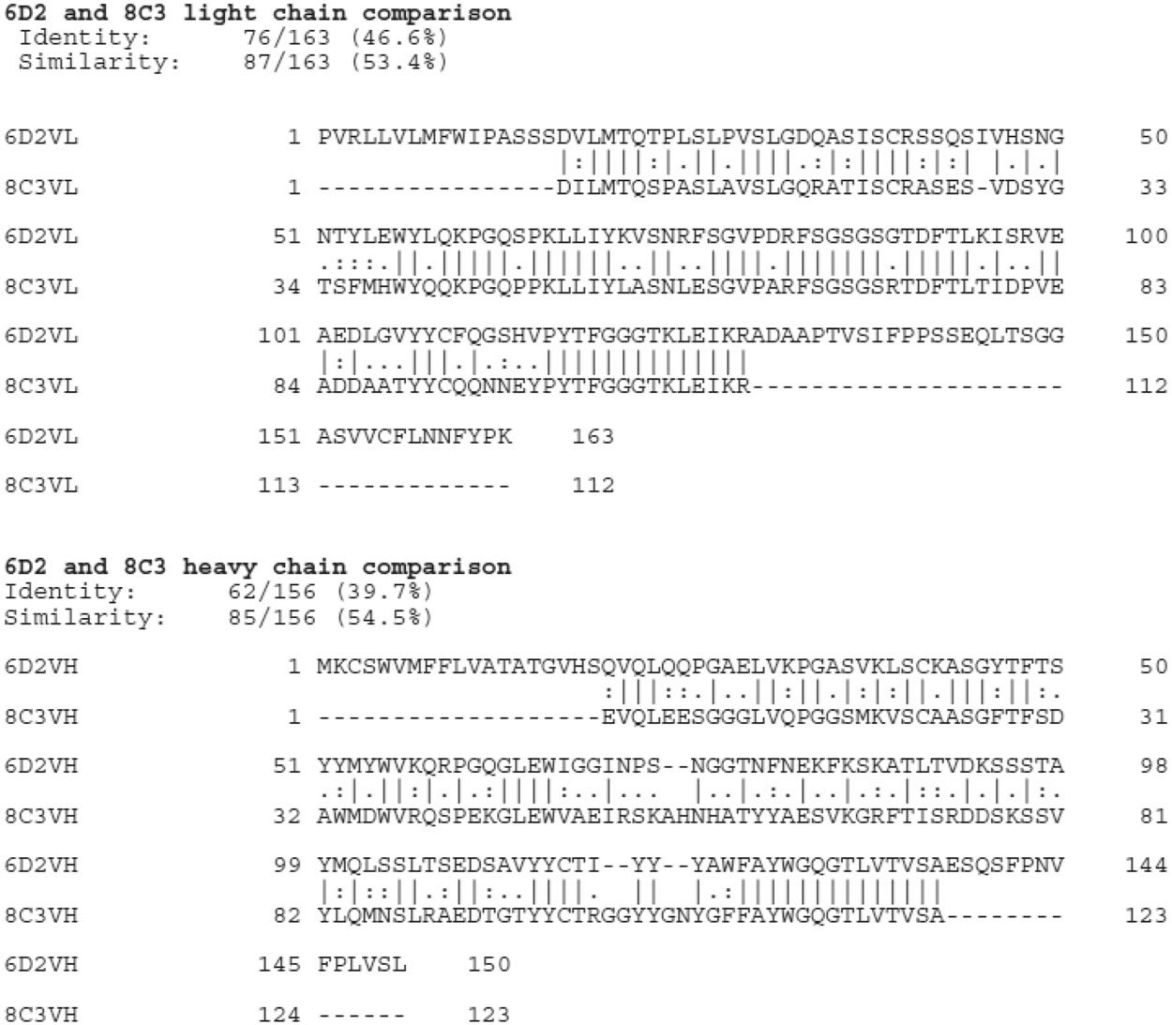
Comparisons of the heavy and light chain protein sequences of mAb 6D2 and 8C3.

The sequences of the IgM 6D2 and IgG 8C3 mAbs were deposited at NCBI GenBank (accession numbers KX346262 and KX346264, respectively). The complementarity determining regions (CDRs) of both mAbs contain high percentage of positively charged amino acids (Fig. 1). This finding suggests that these V regions were selected in part by negative charges on the fungal melanin molecules^28,29^ and that electrostatic attractions contribute significantly to the binding of the mAbs to melanin. Overall, blast analysis showed that the two mAbs have 53% similarity and 47% identity for the VL and 55% and 40% for the VH. Both mAbs have a remarkable abundance of aromatic residues.

**Figure 2:**
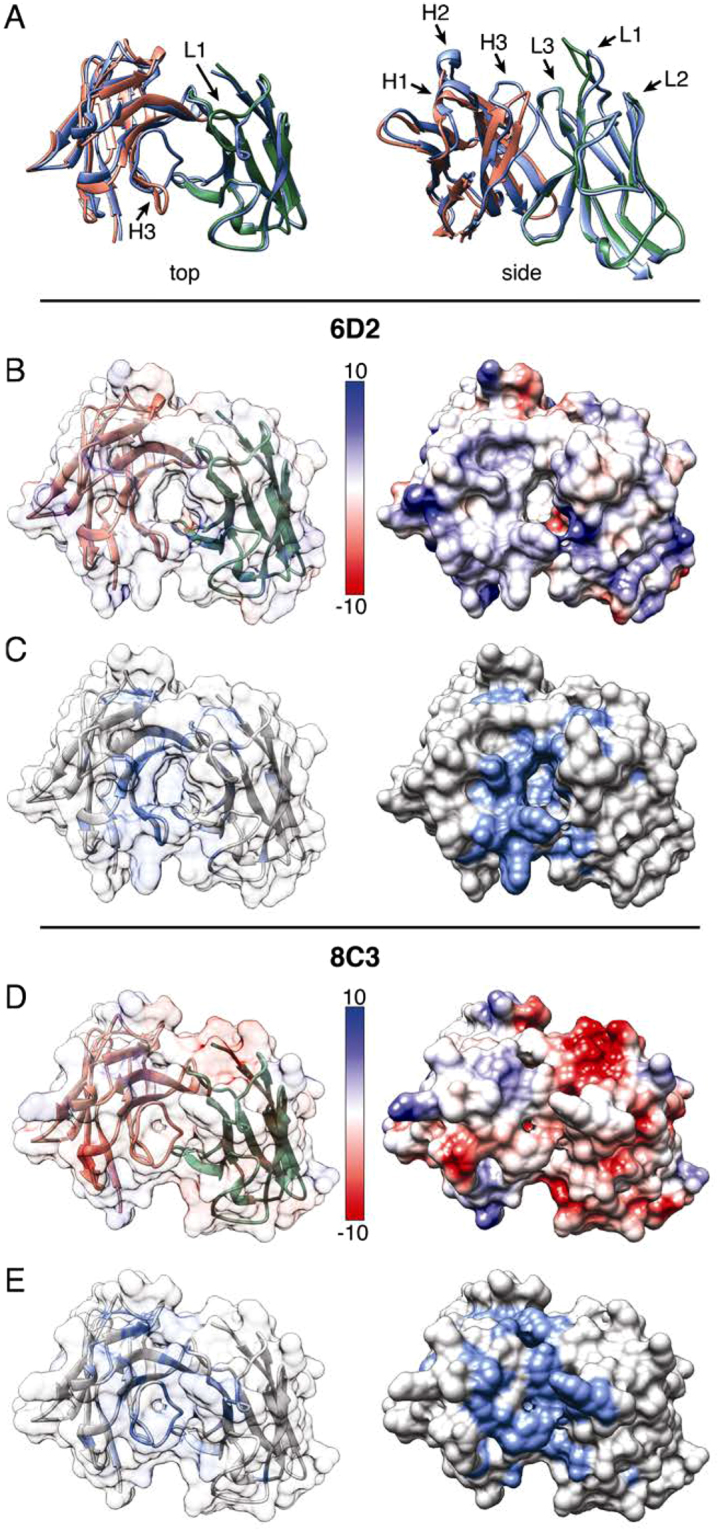
Comparison of 6D2 and 8C3 variable region molecular models. (**A**) Top and side views of the structural alignment of the 6D2 and 8C3 variable regions. 6D2 VH is red, 6D2 VL is green, and both 8C3 chains are blue. CDRs 1–3 on both the H and L chains are labeled in the side view, while the CDR-L1 and CDR-H3 are highlighted in the top view. (**B**) A top view of the surface charge of 6D2 is illustrated by electrostatic potential with red indicating negatively charged regions and blue indicating positively charged regions. The transparent surface view shows the underlying H chain (red) and L chain (green). (**C**) A top view of 6D2 showing aromatic residues highlighted in blue. (**D**) A top view of the surface charge of 8C3 is illustrated by electrostatic potential with red indicating negatively charged regions and blue indicating positively charged regions. The transparent surface view shows the underlying H chain (red) and L chain (green). (**E**) A top view of 8C3 showing aromatic residues highlighted in blue.

We further compared the structures of 6D2 IgM and 8C3 IgG via molecular modeling which revealed additional contributions to mAbs affinity to melanin. Molecular models of the 6D2 and 8C3 V regions were generated based on the most similar known H and L chain antibody structures (Fig. 2). Alignment of these models revealed pronounced structural differences in the CDR L1 and CDR H3 loops (Fig. 2A). Visualization of the 6D2 binding surface indicated a slightly positively charged paratope with a negatively charged pocket (Fig. 2B). The 6D2 paratope also contains several aromatic residues, with most located in CDRs L1, L3, H1, and H3 (Fig. 2C). The 6D2 CDR H3 contains aromatic residues Tyr107, Tyr108, Tyr109, Trp111, and Phe112. The 8C3 model has a negatively charged binding surface, mostly originating from residues in and near the 8C3 VL CDRs: Asp1, Glu27, Asp30, Glu109 (Fig. 2D). There are, however, some notable regions of shared positive charge between 8C3 and 6D2 including CDR H2 and several framework regions. The 8C3 model also shows a highly aromatic paratope from residues in CDRs L1, L3, H1, and H3 (Fig. 2E). The CDR H3 in particular contains aromatic residues Tyr109, Tyr110, Tyr113, Phe115, and Phe116.

**Figure 3:**
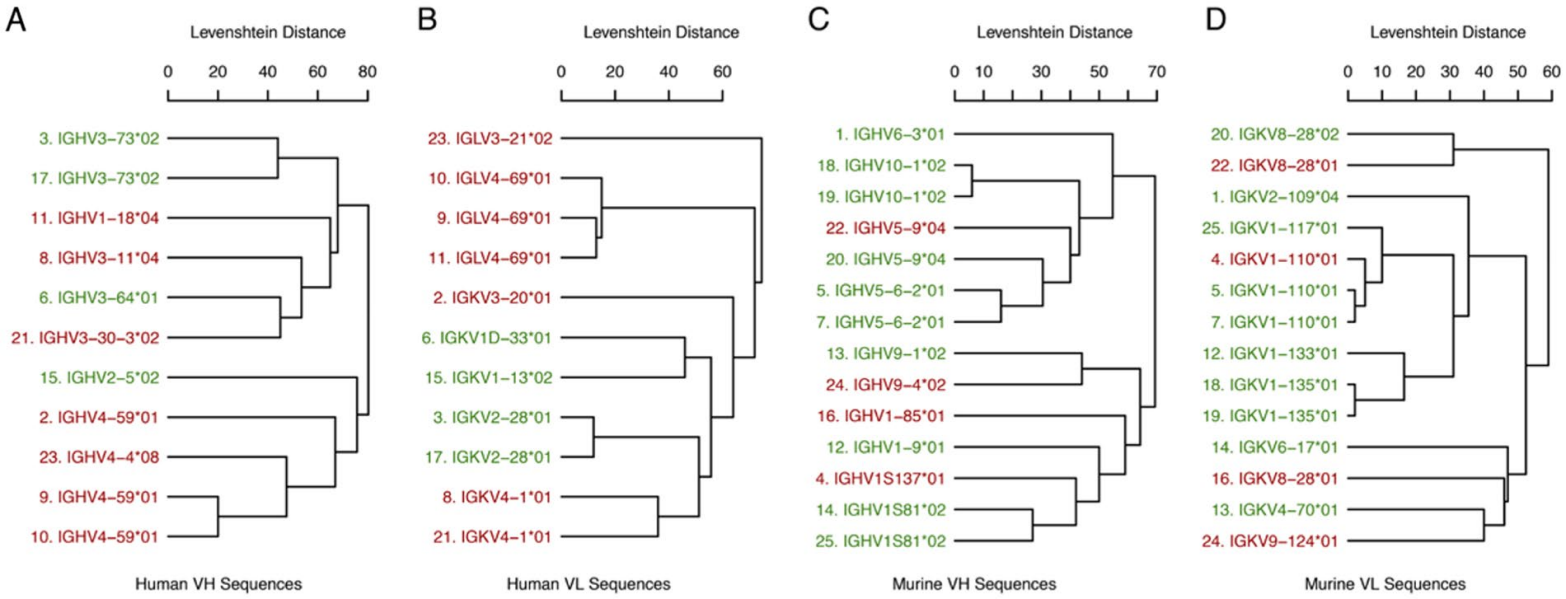
Similarity of permissive and non-permissive antibody sequences with the 6D2 V region sequences. Unique VH (**A**) and VL (**B**) amino acid sequences for 13 of these murine antibodies were described previously^30^. Leaf labels in the dendrograms are colored according to whether changes in the constant region for that antibody were permissive (green) or non-permissive (red) of specificity changes. Numbering of the sequences corresponds to their numbering in the previous review^30^.

Altering the immunoglobulin constant region while leaving the variable region unchanged can cause changes in binding affinity and specificity. A recent review of this phenomenon found that antibodies described in the literature whose binding specificities changed after alterations to the C region clustered around similar V region gene families^30^. While the number of unique murine antibodies in that analysis was relatively small (n = 13), we repeated the phylogenetic clustering here including the 6D2 V region sequences (Fig. 3). The 6D2 sequences are labeled and the numbering of the other sequences corresponds to the numbering in the previous review. The grouping of the 6D2 sequences is consistent with the previous grouping and with the notion that certain V region gene families may differ in being permissive of specificity changes following alteration of the C region. However, firm conclusions cannot be drawn at this time due to the small size of the sample and the limited number of non-permissive sequences.

### IgG 8C3 bound to tumor melanin *in vivo* and did not penetrate healthy melanized tissues

**Figure 4:**
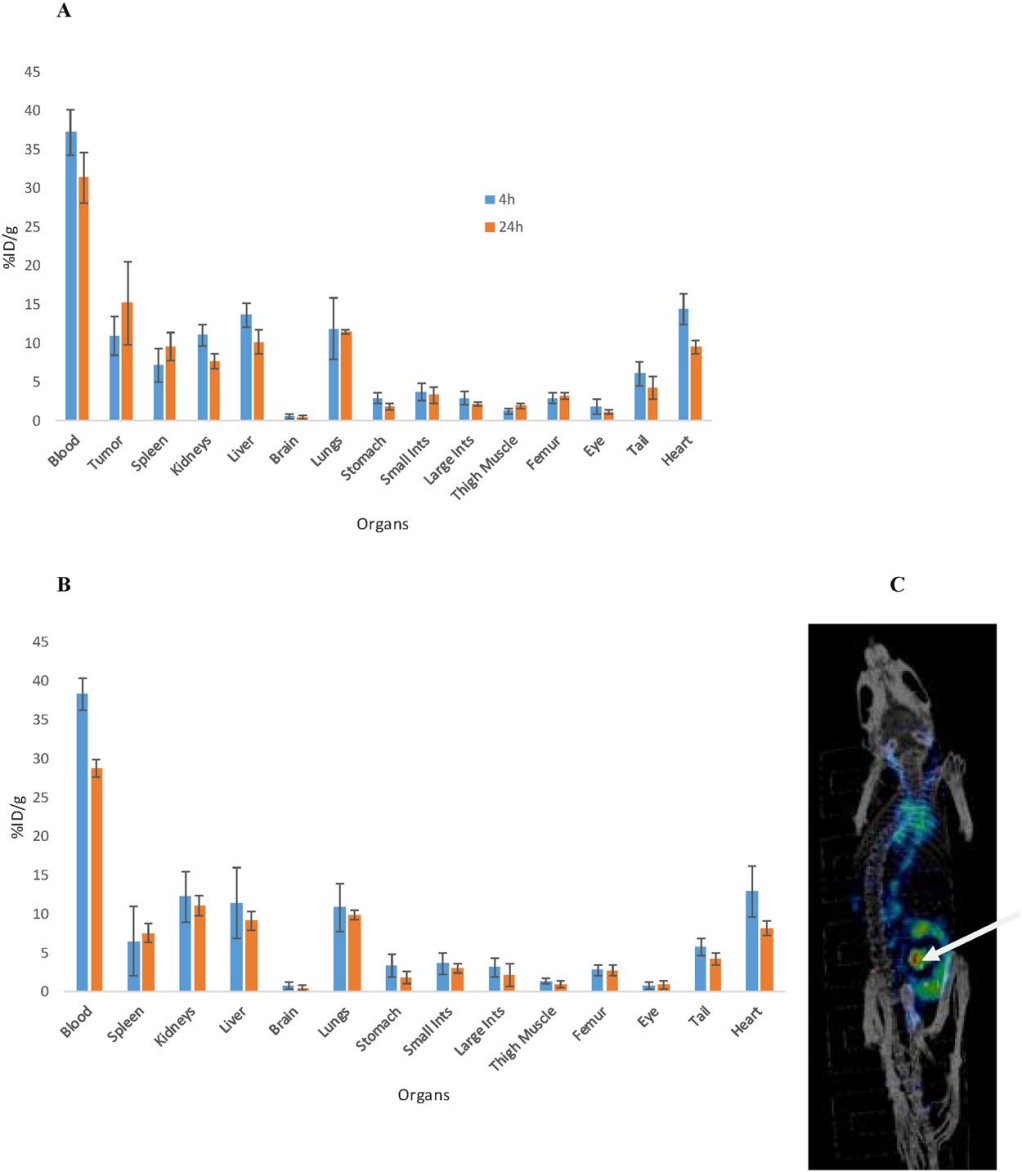
Biodistribution and microSPECT/CT imaging of ^111^In-8C3 in B16-F10 subcutaneous melanoma bearing C57BL6 mice and biodistribution of the rrelevant control ^111^In-IgG in healthy C57BL6 mice after IV administration of the mAbs. The biodistibution was performed at 4 and 24 hrs and microSPECT/CT imaging at 72 hrs. (**A**) ^111^In-8C3 in tumor bearing mice; (**B**) irrelevant control ^111^In-IgG in healthy C57BL6 mice; (**C**) microSPECT/CT of a tumor bearing mouse. The arrow is pointing to the tumor.

The biodistribution of ^111^In-8C3 in B16-F10 melanoma bearing mice demonstrated high uptake, and significantly, in contrast to other tissues, accumulation of the mAb in the tumor which reached 15% ID/g at 24 hrs post ^111^In-8C3 administration (Fig. 4A). The mAb started to clear from the blood-rich organs at 24 hrs and, in general its biodistribution pattern was typical for a murine IgG. Importantly, the uptake of ^111^In-8C3 into the melanin-containing healthy tissues such black eyes and melanized skin on the tails of C57BL6 mice was negligible which demonstrates that this mAb cannot penetrate the membranes of the intact cells and, therefore, cannot damage healthy melanized tissues. To compare the uptake of the melanin-binding antibody into the skin/hair follicles and any ectopic melanin deposits we have compared the uptake of ^111^In-8C3 to that of the irrelevant control IgG (Fig. 4B). The uptake of the irrelevant antibody into the melanized tail was equal to that that melanin-binding 8C3 (Fig. 4B). We have also performed the microSPECT/CT imaging of the B16F10 tumor-bearing mice 72 hr post injection of ^111^In-8C3 - no uptake into the hair follicles, eyes, brain or anywhere in the body except for the tumor and residual activity in blood pool was seen on the microSPECT/CT data (Fig. 4C).

### RIT with radiolabeled 8C3 was effective in reducing melanoma lesions in the lungs and compared favorably to immunotherapy

**Figure 5:**
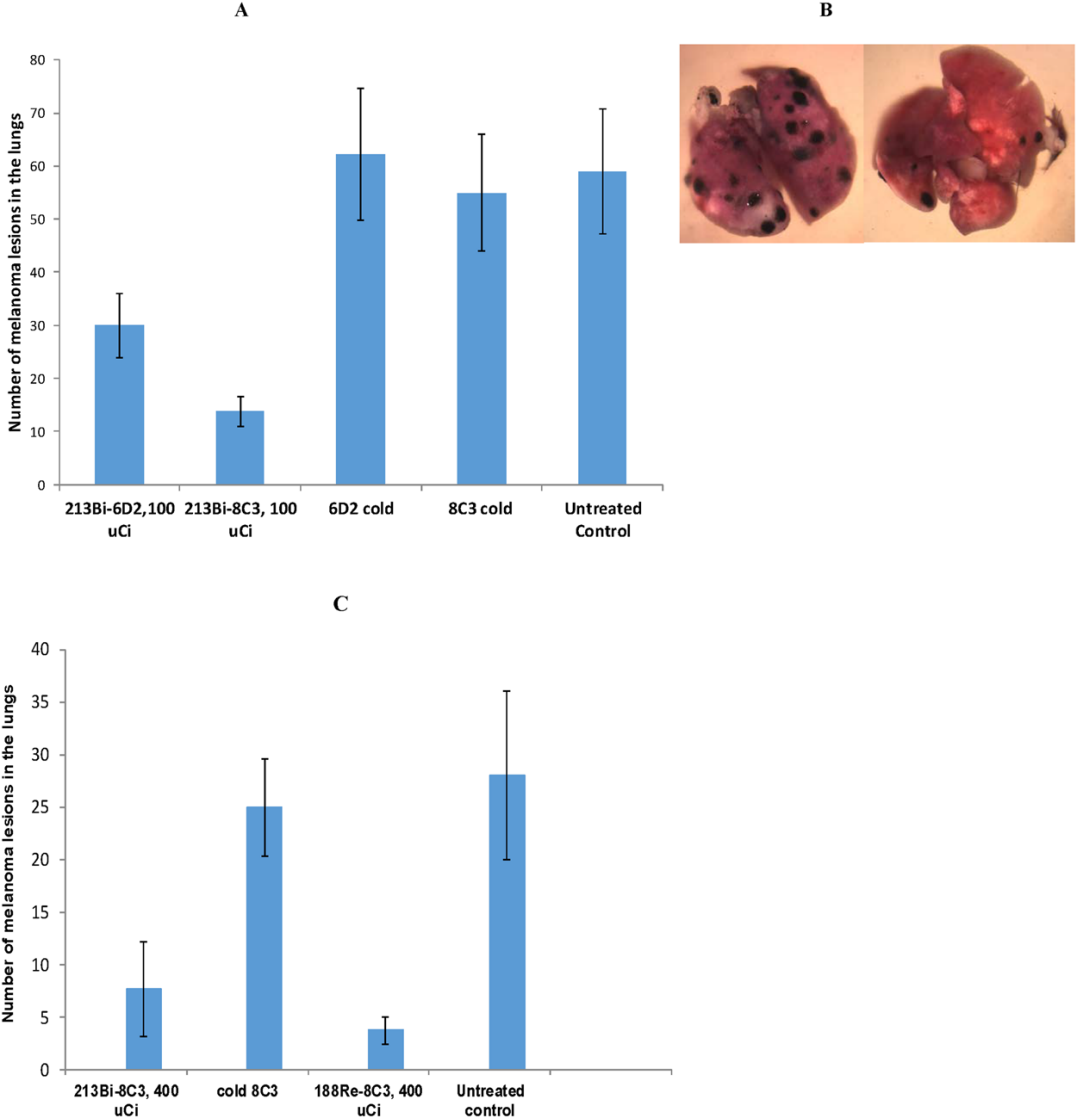
Treatment of B16-F10 metastatic melanoma tumor-bearing C57BL6 mice with radiolabeled 6D2 and 8C3 melanin-binding mAbs. The mice were given B16-F10 murine melanoma cells intravenously via tail vein, treated on Day 4 with the radiolabeled mAbs and sacrificed on Day 14: (**A**) comparative treatment with 100 µCi ^213^Bi-6D2 and ^213^Bi-8C3; (**B**) high resolution microphotographs of the lungs of C57BL6 mice at the conclusion of the experiment. Left panel – unlabeled (“cold”) 8C3 group, right panel - ^213^Bi-8C3 mAb group; (**C**) comparative treatment with ^188^Re-8C3 and ^213^Bi-8C3. ^213^Bi-6D2 or ^213^Bi-8C3 mAbs demonstrated significant (p < 0.01) reduction in metastases-like nodules compared to untreated mice or those administered unlabeled 6D2 or 8C3. ^213^Bi-8C3 was significantly more effective than ^213^Bi-6D2 (p = 0.03). Both ^213^Bi-8C3 and ^188^Re-8C3 achieved significance compared to untreated or unlabeled 8C3 controls (p < 0.01) in treatment of B16-F10 melanoma. ^188^Re-8C3 appeared to be more effective than ^213^Bi-8C3, however, this trend did not reach statistical significance (p = 0.06).

**Figure 6:**
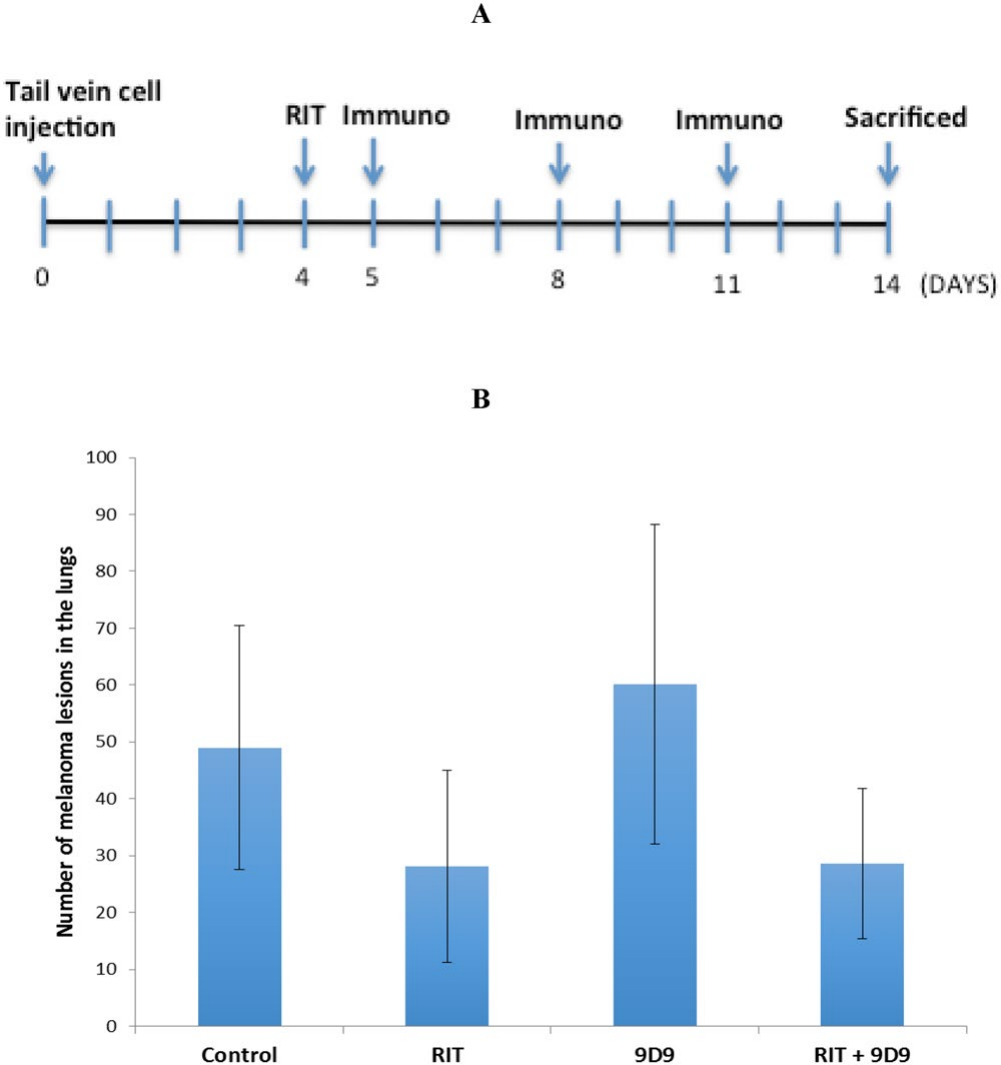
Treatment of B16-F10 metastatic melanoma tumor-bearing C57BL6 mice with RIT delivered by ^213^Bi-8C3, immunotherapy with anti-CTLA4 mAb 9D9 and their combination. (**A**) treatment schematic; (**B**) treatment results. RIT alone and combination treatment groups received IP 150 μCi ^213^Bi-8C3; immunotherapy alone and combination treatment groups received IP 100 μg 9D9 mAb on days 5, 9 and 11 after cell injection. Both the RIT and RIT plus immunotherapy groups showed significantly fewer lung metastases in comparison with the untreated control group (p = 0.045). No statistical difference between the efficacy of RIT alone and the combination of RIT plus immunotherapy groups was observed (p = 0.956).

Treatment of B16-F10 tumor-bearing C57BL6 mice with ^213^Bi-6D2 or ^213^Bi-8C3 mAb demonstrated significant (p < 0.01) reduction in metastases-like nodules in the lungs of mice compared to untreated mice or those administered unlabeled 6D2 or 8C3 (Fig. 5A). The IgG Mab ^213^Bi-8C3, however, was significantly more effective than the IgM Mab ^213^Bi-6D2 in eliminating melanoma lesions in the lungs (p = 0.03) (Fig. 5A). This might be due to better penetration and accumulation in the tumor mass by the IgG antibody. The positive therapeutic results were not only quantitatively significant, but visually impressive. Figure 5B shows the microphotographs of the lungs from a mouse given unlabeled 8C3 mAb (left panel) and a mouse treated with ^213^Bi-8C3 mAb. Importantly, the radiolabeling of 8C3 with ^213^Bi was accomplished with the high radiolabeling yield of ≥ 95% and required no post radiolabeling purification. In contrast, ^213^Bi-6D2 required extensive purification from unreacted ^213^Bi, further emphasizing advantages of developing the IgG isotype over that of the IgM for RIT. The comparison of the beta- versus alpha-emitting radiolabel for RIT demonstrated practically the equivalence of ^213^Bi-8C3 and ^188^Re-8C3 efficacy, both achieving significance compared to untreated or unlabeled 8C3 controls (p < 0.01) in the metastatic B16-F10 melanoma model (Fig. 5C). ^188^Re-8C3 appeared to be more effective than ^213^Bi-8C3, however, this trend did not reach statistical significance (p = 0.06). Finally, we compared ^213^Bi-8C3 RIT with anti-CTLA4 immunotherapy as well as investigated the effect of their combination on the metastasis-like B16-F10 lesions in mouse lungs. The time line of the treatment is shown in Fig. 6A. These results demonstrate that anti-CTLA-4 mAb 9D9 alone could not contain the growth of tumors in this aggressive melanoma model. Both the RIT and RIT plus immunotherapy groups showed significantly fewer lung metastases with n = 28 (±16.9) and n = 28.6 (±3.2), respectively, in comparison with the untreated control group (p = 0.045). However, there was no statistical difference between the efficacy of RIT alone and the combination of RIT plus immunotherapy groups (p = 0.956), suggesting that the effect on the tumor in tumor in the combination treatment group may have resulted solely from RIT.

## Discussion

Targeted radionuclide therapy of melanoma is experiencing a resurgence^31^ and can be offered to patients who are not responding to current standard of care immunotherapies or used in concert with the immunotherapy. This prompted us to investigate the structure-function relationship and the therapeutic potential of an IgG mAb to fungal melanin generated in our laboratories.

Although immunization of mice with fungal melanin particles elicits an Ab response that includes IgM and IgG, the mechanism by which the immune system recognizes insoluble highly charged melanin particles is unknown^32^. Only a few mAbs to melanin to have been reported, of which mAb 6D2 is the best characterized. This mAb helped to prove that *C. neoformans* melanizes *in vivo*^23^, demonstrated that Abs to melanin have antifungal properties^33^, and subsequently was utilized in melanoma RIT^24–26^. The high number of aromatic amino acids in the CDR regions suggests that these residues could potentially interact with melanin through aromatic interactions since melanin pigment is composed of cross-linked subunits with high aromatic content^34,35^. Given melanin’s negative charge, it is likely that aromatic interactions predominate for 8C3 binding. The structural differences between the two models also suggest different melanin epitopes for the two mAbs. Compared to 6D2, the highly aromatic CDR H3 loop of 8C3 is flipped toward the center of the binding groove, blocking an interior pocket present in the 6D2 structure. Additionally, the 8C3 CDR L1 loop is pulled away from the binding groove but contains the addition of aromatic residues Phe38 along the paratope edge and Tyr34, which protrudes above and back toward the paratope. In contrast, the 6D2 CDR L1 lines the wall of the binding groove with aromatic residues His31, Tyr38, and His109.

The IgG isotype of 8C3 facilitated quantitative radiolabeling with ^213^Bi and ^188^Re, which is in striking contrast with the IgM 6D2 used in previous pre-clinical and clinical work, which required extensive post-radiolabeling purification. The ability of radiolabeled 8C3 to bind melanin *in vivo* clearly resulted from its structure and its many common features with the 6D2 IgM to melanin. Both ^213^Bi and ^188^Re radionuclides transformed the 8C3 mAb into a therapeutic agent with an impressive efficacy in the very aggressive B16-F10 melanoma model. Importantly, 8C3 was not taken up by the healthy melanin containing tissues such as melanized skin or hair follicles due to its inability to cross the cellular membrane and access intracellular melanin. This important feature will ensure its safety towards healthy tissues when used for radioimmunotherapy. Performing this safety evaluation was important, because melanoma cells often secretes the pigment into the environment, where it can be phagocyted by macrophages, or even some melanoma cells may undergo fusion with macrophages. Macrophages may spread the tumor-produced melanin around the organism which created additional danger for normal tissues^16,36–38^. In our future evaluation of the safety of RIT with the humanized version of 8C3 mAb to melanin we are planning to utilize a model of cyclophosphamide-induced alopecia in C57BL6 mice described in^38^ which can damage hair follicles and make melanin accessible to the 8C3 mAb, thus emulating a situation of a melanoma patient treated with chemotherapy before being treated with RIT. Effectiveness of ^213^Bi-8C3 mAb is particularly encouraging as the interest in, and the use of targeted systemic oncologic therapies with high relative biological effectiveness (RBE) alpha-particle emitters is currently burgeoning worldwide^11–14^.

As part of the evaluation of 8C3 mAb as a potential RIT agent, we performed its side by side comparison with one of the standards of care drugs for metastatic melanoma treatment – anti-CTLA4 mAb. For this we utilized anti murine-CTLA4 mAb 9D9 which has been tested previously in the B16F10 murine melanoma model^39,40^. In addition, we investigated if preceding the immunotherapy with RIT would increase the efficacy of the former, potentially via an additive or synergistic effect. In this regard, combination treatments have been performed in the treatment of multiple myeloma using alpha-RIT followed by adoptive T-cell transfer (ACT), and results have demonstrated a significant decrease in tumor size compared to control mice or mice that received RIT alone, and those that received ACT alone^41^. The results of our experiment demonstrated that while RIT was quite effective in reducing the melanoma nodules in the lungs of the mice, no reduction was observed in anti-CTLA4 mAb group. The latter result was somewhat expected, since prior studies using the same B16F10 model had demonstrated that the anti-CTLA4 mAbs alone were not very effective^39,40^. Similarly, in clinical practice, though anti-CTLA-4 mAb ipilimumab has been approved as a single agent treatment for patients with metastatic melanoma, currently it is primarily being used in combination with other agents including anti-PD-1 antibodies, anti-cancer vaccines, chemotherapy and radiation^6,7,42,43^. Interestingly, in these studies we were unable to demonstrate that preceding anti-CTLA-4 mAb administration with RIT provided a boost to immune system evidenced by the lack of reduction in the number of lesions in the combination therapy group. This was, most likely, due to the remarkable efficacy of RIT alone and the complete lack of anti-CTLA4 mAb efficacy. In this regard, anti-CTLA4 therapy often needs cooperation from the host microbiome which might not be present in the mice from every vendor^44^. Taken together, these results of the side by side comparison of RIT with anti-CTLA4 immunotherapy demonstrate the advantages of RIT and emphasize its independence of the immune status of the host or state of its microbiome. We hope that future experiments on combining RIT with anti-PD1 and/or anti-PDL-1 mAbs might produce synergistic results.

In conclusion, two mAbs to fungal melanin - IgM 6D2 and IgG 8C3 - generated a decade apart, showed remarkable similarity in their variable region and CDR as well as an abundance of aromatic residues that may influence their capacity to bind melanin. The encouraging preliminary results on using 8C3 as a targeting molecule for RIT of experimental metastatic melanoma provide the impetus for the development of melanin-targeted IgG isotype mAbs for clinical applications in treatment of metastatic melanoma. Future attempts to generate chimeric Abs or humanize the murine melanin-binding Abs need to consider that these V regions are permissive to C-mediated changes, suggesting that additional engineering would be required to insure that the murine-derived Vs bind melanin.

## Materials and Methods

### Data availability statement

The antibodies sequences are available at NCBI GenBank (accession numbers KX346262 and KX346264). The authors will make materials, data and associated protocols promptly available to readers upon publication in Scientific Reports without undue qualifications in materials transfer agreements.

### mAbs, cell lines and radionuclides

Melanin-binding 6D2 IgM was produced by Goodwin Biotechnologies (Plantation, FL, USA) as described in Dadachova *et al*.^24^ and 8C3 IgG was produced in our laboratories as described in Urán *et al*.^27^. Anti-murine CTLA4 mAb 9D9 for the immunotherapy treatment arm was purchased from BioXCell (West Lebanon, NH, USA) and IgG control mAb – from Creative Diagnostics (NYC, NY, USA). The pigmented murine melanoma cell line B16-F10 was obtained from American Type Culture Collection (Manassas, VA, USA). 213-Bismuth (^213^Bi), the alpha particle emitting radionuclide, was eluted from the ^225^Ac/^213^Bi radionuclide generator system, produced by the Directorate for Nuclear Safety and Security, Karlsruhe, Germany. 188Rhenium (^188^Re) beta emitter was eluted from the ^188^W/^188^Re radionuclide generator (ITG, Garching, Germany). 111Indium (^111^In) in form of ^111^In chloride was purchased from MDS Nordion (Vancouver, BC, Canada). The CHXA” bifunctional chelating agent for labeling the mAbs with ^111^In and ^213^Bi was obtained from Macrocyclics (Plano, TX, USA).

### Sequencing of 6D2 IgM and 8C3 IgG1

6D2 sequencing was performed by Fusion Antibodies Ltd (Belfast, Northern Ireland). 6D2 mRNA was extracted from the cell pellets using Fusion Antibodies in-house RNA extraction protocol. 8C3 was sequenced at the Albert Einstein College of Medicine from cDNA reverse transcribed from the RNA using an oligo(dT) primer. PCR reactions were designed to amplify the variable regions of the cell line. The constant and variable region PCR products were cloned into the sequencing vector pCR2.1 (Invitrogen) for 6D2 or into pGEM T easy vector (Promega) for 8C3, and then transformed into TOP10 *Escherichia coli* (Thermo Fisher Scientific) for the selection of positive transformants. Selected colonies were picked and analyzed through sequencing by both vbase2 (http://www.vbase2.org/vbase2.php) and NCBI blast (http://www.ncbi.nlm.nih.gov/igblast/). Sequences of the mAbs were compared by Clustal Omega (http://www.ebi.ac.uk/Tools/psa/emboss_needle/).

### Molecular modeling of 6D2 IgM and 8C3 IgG

Nucleotide sequences for the H and L chains of 6D2 and 8C3 were analyzed using version 3.4.0 of the IMGT/V-QUEST tool for murine immunoglobulin sequences^45^. This tool was used to identify the closest germline V region gene for each sequence and infer the V region family. The IMGT unique numbering scheme reported by the IMGT/V-QUEST tool was used when referring to the residue numbers in the molecular models. The translated amino acid sequence of each V region was used to generate structural models of 6D2 and 8C3 using the Prediction of Immunoglobulin Structure (PIGS) server^46^. PIGS models were generated based on the best H and L chain templates from known structures. CDR loops were modeled based on loops with similar canonical structure, while CDR H3 was grafted. Molecular graphics, structural alignment of models, and calculation of surface electrostatic potential were performed with UCSF Chimera, which is developed by the Resource for Biocomputing, Visualization, and Informatics at the University of California, San Francisco (supported by NIGMS P41-GM103311)^47^. Highlighted aromatic residues consisted of Phe, Tyr, Trp, and His.

We also compared the V region elements of the melanin Abs to sequences of Abs that had shown changes in specificity when the C region was changed (permissive) and those that had not (non-permissive). A dendrogram showing the similarity of 6D2 antibody V region sequences with other permissive and non-permissive antibody sequences was created as previously described^30^. Briefly, immunoglobulins identified in the literature with differing C regions and identical V regions were grouped according to whether or not specificity changes were observed after altering the constant region. Germline genes for each antibody were identified using the IgBLAST sequence analysis tool^48^. Dendrograms were constructed through hierarchical average-linkage clustering with pairwise sequence similarity calculated as the Levenshtein or edit distance. The 6D2 V region sequences were included in this analysis while the other 13 murine sequences are identical to those described in the previous review^30^.

### B16-F10 melanoma mouse models

All animal experiments were performed according to the protocols approved by the Institute of Animal Studies at the Albert Einstein College of Medicine (#20151106) and by the Animal Research Ethics Board of the University of Saskatchewan (#2017006). For the biodistribution experiment, 5 million B16-F10 murine melanoma cells suspended in matrigel were injected subcutaneously with into the right flank of 6 week old C57BL6 female mice (Charles River Laboratories, Wilmington, MA). The biodistribution analysis was performed on Day 8 post tumor cells injection when the tumors reached 0.7–1.0 cm in diameter. For therapy experiments, C57BL6 mice were injected intravenously through the tail with 2 hundred thousand B16-F10 cells/200 μL and treated with RIT and/or immunotherapy 4 days after tumor cells inoculation. In this model the melanoma lesions form in the lungs in form of microcolonies. This model for lung colonization in melanoma and testing of the drug conjugates was introduced by Overwijk and Restifo in 2001^49^ and has been widely used in melanoma field since then^50^.

### Biodistribution and microSPECT/CT of ^111^In-8C3 mAb in subcutaneous B16-F10 melanoma

The 8C3 and control IgG mAbs were conjugated to the bifunctional chelator CHXA” and radiolabeled with ^111^In as described in McFarren *et al*.^51^. The resulting specific activity was 2 µCi/µg mAb. The mice were injected intravenously (IV) via tail vein with 33 µCi ^111^In-8C3 or ^111^In-IgG in 100 µL PBS. At the predetermined time points of 4 and 24 hrs the groups of 5 mice were humanely sacrificed, their tumors and major organs removed, blotted from blood, weighed, counted for radioactivity in a gamma counter, and percentage of injected dose per gram (ID/g) organ/tissue was calculated. For microSPECT/CT (micro single photon emission computer tomography/computer tomography) imaging some of the tumor-bearing mice were injected IV with 200 µCi ^111^In-8C3 in 100 µL PBS and imaged 72 hrs post administration. The imaging was performed on microSPECT/CT (VECTor4CT, Utrecht, NL).

### Evaluation of the radiolabeled 8C3 IgG as an RIT agent in murine melanoma model and its comparison with immunotherapy

In the first series of therapy experiments the efficacy of 8C3 mAb radiolabeled with ^213^Bi as described in McFarren *et al*.^51^ was compared with that of 6D2 mAb. The mAbs were radiolabeled with 100 µCi ^213^Bi and administered intraperitoneally to C57BL6 mice on Day 4 post B16-F10 intravenous tumor cells injection. Mice treated with unlabeled (“cold”) 8C3 and 6D2 mAbs as well as untreated mice served as control groups. The mice were sacrificed on Day 14 post tumor cell injection and the metastases-like melanoma lesions in their lungs were enumerated with the 20 x −40 x Binocular Stereo Dissecting Microscope with USB Camera (AmScope). In the second series of experiments the efficacy of ^213^Bi- and ^188^Re-labeled 8C3 was compared. For this purpose 8C3 was radiolabeled with 200 and 400 µCi ^213^Bi and ^188^Re as described in McFarren *et al*.^51^ and Dadachova *et al*.^25^, respectively, and mice were treated on Day 4 post tumor cells injection as described above. Finally, in the third series of experiments we compared the efficacy of RIT with ^213^Bi-8C3 to that of immunotherapy with anti-CTLA4 mAb 9D9. The four groups included untreated mice, RIT alone, immunotherapy with anti-CTLA4 9D9 mAb alone, and the combination of RIT and immunotherapy. On day 4 after cell injection, the RIT alone and combination treatment groups received 150 μCi/100 μL ^213^Bi-8C3 as intraperitoneal (IP) injection. The same volume of PBS was given IP to the untreated control group. On days 5, 9 and 11 after cell injection, the immunotherapy alone and combination treatment groups received IP injection of 100 μg/100 μL of 9D9 mAb following the treatment protocol described in Curran *et al*.^39^.

### Statistical analysis

Power was estimated using PASS version 11 (NCSS, Inc., Kaysville, UT, http://www.ncss.com) using simulations of different tumor volumes based on our pilot data and conservative assumptions regarding the groups treated with the radiolabeled antibodies. All simulations showed power of at least 83% with only five animals per group because of the large differences between treated and untreated animals. Thus, 5 mice per group were utilized in all studies. The differences between the groups was analyzed using Kruskal-Wallis test.
